# Research progress and application of liver organoids for disease modeling and regenerative therapy

**DOI:** 10.1007/s00109-024-02455-3

**Published:** 2024-05-28

**Authors:** Yang Hu, Qiao Geng, Lu Wang, Yi Wang, Chuyue Huang, Zhimin Fan, Desong Kong

**Affiliations:** 1 Chinese Medicine Modernization and Big Data Research Center, Nanjing Hospital of Chinese Medicine affiliated to Nanjing University of Chinese Medicine, Nanjing University of Chinese Medicine, Nanjing, 210022 Jiangsu China; 2 Department of Pharmacology, School of Pharmacy, Nanjing University of Chinese Medicine, 138 Xianlin Avenue, Nanjing, 210023 China; 3 Department of Angioenterology, Nanjing Hospital of Chinese Medicine affiliated to Nanjing University of Chinese Medicine, Nanjing University of Chinese Medicine, 157 Daming Avenue, Nanjing, 210022 Jiangsu China

**Keywords:** Liver, Organoids, 3D culture, Disease model, Application

## Abstract

The liver is a major metabolic organ of the human body and has a high incidence of diseases. In recent years, the annual incidence of liver disease has increased, seriously endangering human life and health. The study of the occurrence and development mechanism of liver diseases, discovery of new therapeutic targets, and establishment of new methods of medical treatment are major issues related to the national economy and people’s livelihood. The development of stable and effective research models is expected to provide new insights into the pathogenesis of liver diseases and the search for more effective treatment options. Organoid technology is a new in vitro culture system, and organoids constructed by human cells can simulate the morphological structure, gene expression, and glucose and lipid metabolism of organs in vivo, providing a new model for related research on liver diseases. This paper reviews the latest research progress on liver organoids from the establishment of cell sources and application of liver organoids and discusses their application potential in the field of liver disease research.

## Overview of the liver

The liver is a primary metabolic organ in animals and is responsible for glycogen synthesis and storage, bile secretion and excretion, synthesis of certain secretory proteins, and detoxification. It consists mainly of parenchymal cells, specifically hepatocytes, and nonparenchymal cells, including hepatic stellate cells (HSCs), Kupffer cells, sinusoidal endothelial cells, bile duct epithelial cells, hepatic dendritic cells, NK cells, and NKT cells [1]. Owing to factors such as dietary habits, viral infections, drug-induced damage, and genetic variations, the incidence of liver-related diseases is increasing, making them a leading cause of mortality worldwide [2–4]. The complex and diverse microenvironment within the liver presents significant challenges for constructing efficient and accurate research models. Currently, research on the pathogenesis of liver-related diseases and drug development primarily relies on in vivo animal models and in vitro two-dimensional (2D) models. However, in vivo animal models are subject to species differences and have long experimental cycles, making them unsuitable for large-scale drug safety evaluation and screening. In vitro 2D models, such as simple monolayer cell culture models, significantly differ from the actual physiological environment within the liver and fail to represent cellular developmental processes in vivo [5, 6]. Therefore, there is an urgent need to establish in vitro research models that can better mimic the human liver and identify more effective treatment approaches. The emergence of novel organoid culture technologies has ignited excitement among researchers worldwide, as they offer cell compositions that closely resemble physiological conditions in the human body and exhibit similar three-dimensional structures, making them a hot topic in research. 

## Overview of the organoids

Organoids are three-dimensional organic structures that resemble the physiological functions and structures of in vivo organs, formed by the self-organization and differentiation of induced pluripotent, embryonic, and adult stem cells in vitro [7, 8]. In 2009, Clevers et al. [9] first reported the construction of natural intestinal organoids. They extracted intestinal stem cells and cultured them in vitro, resulting in organoids with intestinal crypts and villus-like structures, sparking the trend in organoid research. In 2013, Clevers et al. [10] reported the successful generation of liver organoids using Lgr5^+^ liver stem cells, which initiated the study of hepatic organoids. In 2017, organic technology was recognized as the Best Method of the Year in the field of life sciences by Nature Methods [11]. After over a decade of research, various organoid models have been successfully established, including the intestines [9, 12, 13], liver [10, 14, 15], pancreas [16, 17], lungs [18–20], stomach [21–23], esophagus [24, 25], bladder [26], kidneys [27–29], ovaries [30, 31], mammary glands [32–34], prostate [35–37], brain [38–40], and heart [41, 42]. Compared with traditional models, organoids can self-organize into three-dimensional structures, simulate the development process and physiological environment of in vivo organs under the regulation of exogenous growth factors, undergo multilineage differentiation, self-organize to form tissue or organ structures similar to the source tissue, and exhibit certain organ functions. Simultaneously, organoids can be massively expanded while maintaining genomic stability [43] and are easily manipulated and suitable for large-scale drug evaluation and screening [44].

## Source of liver organoids

Liver organoids can be classified based on different cell sources, including induced pluripotent stem cells (iPSCs), embryonic stem cells (ESCs), adult stem cells (ASCs), and patient-derived organoids (PODs) from tumor cells.

### PSC-derived liver organoids

#### iPSC-derived liver organoids

**Fig. 1:**
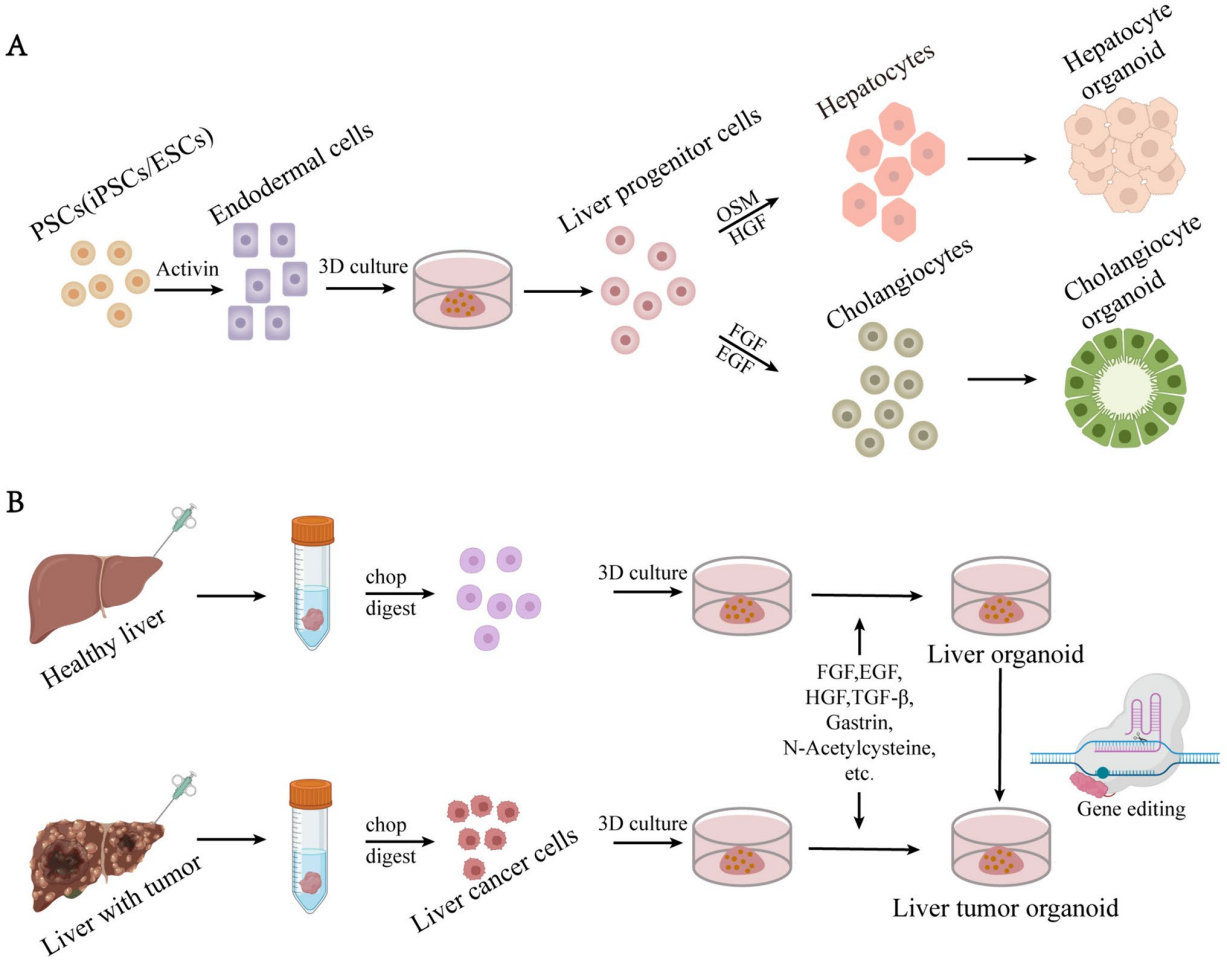
The construction of liver organoids. The construction process of liver organoids derived from PSC **A**. The construction process of liver organoids derived from ASC and liver cancer cell **B**

**Fig. 2:**
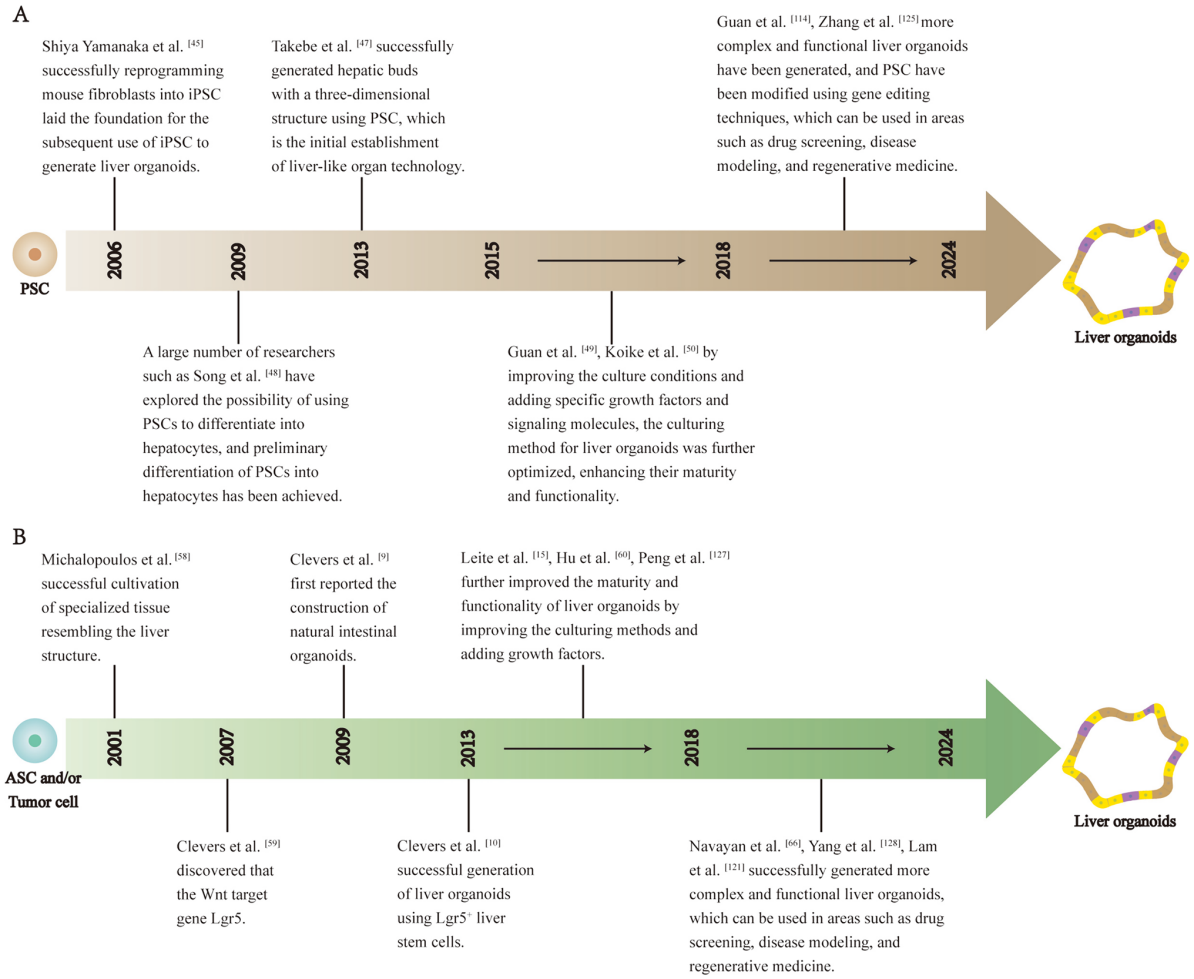
The development of liver organoids. The development of liver organoids derived from PSC **A**. The development of liver organoids derived from ASC and liver cancer cell **B**

Induced pluripotent stem cell (iPSC) technology refers to the reprogramming of terminally differentiated somatic cells into pluripotent stem cells by the introduction of specific transcription factors. iPSCs were initially developed by Japanese scientist Shinya Yamanaka [45] in 2006, who used a viral vector to introduce four transcription factors (Oct3/4, Sox2, Klf4, and c-Myc) into differentiated mouse fibroblasts. This reprogramming resulted in a cell type closely resembling embryonic stem cells in terms of cellular morphology, gene and protein expression, epigenetic modifications, cell proliferation capacity, and differentiation potential. iPSCs have a wide range of sources and possess the developmental potential of early embryonic stem cells. They can be cultured in suitable systems in vitro to mimic the developmental processes that occur in vivo and can differentiate into various adult cell types and tissues. Additionally, the use of iPSCs avoids the ethical concerns associated with embryonic stem cells [46]. Because of these characteristics, iPSCs have tremendous potential for biomedical research and development (Figs. 1 and 2).

In 2013, Takebe et al. [47] successfully co-cultured iPSC-derived endodermal cells with human umbilical vein endothelial cells and human mesenchymal stem cells in a 3D cell culture matrix, resulting in the formation of liver bud cell clusters similar to those in human liver tissue. These liver bud clusters exhibited similar functionality as liver buds in vivo when transplanted into immunodeficient mice. Prior to this, a large number of researchers such as Song et al.[48] explored the possibility of using PSCs to differentiate into hepatocytes and initially achieved the differentiation of PSCs into hepatocytes by optimizing culture conditions and growth factors. In 2017, Guan et al. [49] generated a complex structure composed of hepatocytes and bile duct cells by using iPSCs. iPSCs differentiate into liver-like organoids in stages similar to embryonic liver development. This study also demonstrated that genomic engineering can introduce disease-causing mutations into iPSCs, allowing detailed analysis of their functions within organoids. In 2019, Koike et al. [50] established a complex organoid model with continuous and dynamic liver-biliary-pancreatic structures using iPSCs, providing theoretical guidance for studying complex boundary interactions and generating interconnected multiorgan structures in personalized human organ models for in vitro organogenesis and disease research. In 2020, Takeishi et al. [51] generated a functional human mini-liver with microscale structures by differentiating human iPSC-derived hepatocytes, endothelial cells, bile duct cells, primary human liver-derived fibroblasts, and mesenchymal stem cells. These mini-livers survived for four days when transplanted into immunodeficient rats. This achievement opens promising prospects for future human-engineered organ cultivation and transplantation. In 2023, Weng et al. [52] successfully developed liver organoids derived from iPSCs in rotating wall vessels (RWVs) without Matrigel, which exhibited greater hepatocyte-specific functionality compared to those formed on Matrigel. RWV liver organoids avoided the detrimental effects of Matrigel on hepatic differentiation, maintained prolonged functionality during long-term culture, and expressed a range of mature functional genes at levels comparable to the adult liver, while retaining some fetal characteristics. RWVs have revolutionized traditional liver organoids culture conditions, offering a simple and high-throughput approach to generating Matrigel-free liver organoids suitable for research and clinical applications.

#### ESC-derived liver organoids

Embryonic stem cells are a type of cells derived from early stage embryos (prior to the gastrulation stage) or primordial germ cells. They possess the characteristics of unlimited proliferation, pluripotency, and self-renewal and can be induced to differentiate into almost all cell types of an organism, both in vivo and in vitro [53]. In 1981, Evans and Kaufman [54] successfully obtained stem cell lines from mouse cheek pouches and achieved their in vitro cultivation. They found that these cells have great potential for unlimited proliferation and multidirectional differentiation. In 1998, Thomson et al. [55] first derived human embryonic stem cell lines from human embryos, using a new era of research on human ESCs. In addition to differentiating into specific mature cells, ESCs can also be induced to form organoids in vitro. In 2019, Wang et al. [56] successfully derived liver organoids from ESCs, which stably maintained the potential for bidirectional differentiation into functional hepatocytes and cholangiocytes. The research results demonstrated that these liver organoids exhibited robust liver regenerative capacity when transplanted into FRG liver injury mice and could differentiate into mature hepatocytes. In 2022, Cheng et al. [57] utilized embryonic stem cells (ESCs) cultured in mTeSR1 medium to induce stepwise hepatic differentiation, constructing liver organoids. Using liver organoids derived from this method, they investigated the hepatotoxic mechanisms of microplastics (MP) and the plasticizer bisphenol A (BPA). The research findings demonstrated that even low doses of simultaneous exposure to MP and BPA posed metabolism-associated risks to the liver.

### ASC-derived liver organoids

Adult stem cells are undifferentiated cells present in differentiated tissues and organs. These cells have the ability to self-renew and differentiate into specific cells that make up their respective tissues or organs. In 2001, Michalopoulos et al. [58] isolated liver and other cells from adult rats using liver collagenase perfusion. These cells were then cultured in collagen-coated flasks using a liver cell growth medium supplemented with hepatocyte growth factor (HGF) and epidermal growth factor (EGF), resulting in the successful cultivation of specialized tissue resembling the liver structure, although their survival time was extremely short. In 2007, Clevers et al. [59] discovered that the Wnt target gene Lgr5 (also known as Gpr49) served as a marker for intestinal stem cells in the small intestine and colon, and its expression pattern could delineate adult tissues and cancer stem cells. In subsequent studies, individual Lgr5^+^ stem cells were expanded into transplantable liver bud organoids in a 3D culture system containing the Wnt agonist RSPO1 [12, 22]. Lgr5^+^ stem cells that are capable of forming organoids are present in both human and mouse adult livers. In 2018, Hu et al. [60] established a long-term culture system for mouse and human primary liver cells to generate hepatocyte organoids. The research revealed that these organoids could be established and maintained for several months from a single hepatocyte while retaining crucial morphological, functional, and gene expression characteristics. The transcription profile of the organoids resembled that of hepatocytes undergoing proliferation after partial hepatectomy. When transplanted into mice with liver injury, the organoids exhibited extensive proliferation, recapitulating the regenerative response of hepatocytes. In 2019, Huch et al. [61] utilized cloning and long-term expansion of murine and human liver ductal cells to generate self-renewing bipotent organoids capable of differentiating into ductal and hepatocyte-like cells both in vitro and in vivo. They also demonstrated that the transcriptional landscape of resident ductal cells undergoes genome-wide changes following liver injury and during liver organoid formation, accompanied by significant reshaping of their DNA methylome and hydroxymethylome. These organoids are essentially composed of epithelial cells, typically lacking a mesenchymal or stromal cellular compartment that can be maintained in culture. Additionally, with passage, there is a loss of resident immune cells as well as a loss of differentiated cell types.

### Tumor cell-derived liver organoids

Tumor heterogeneity refers to the presence of cells with different genes and phenotypes within the same tumor tissue or between different tumor tissues [62]. This characteristic is a key factor contributing to cancer treatment failure [63, 64]. Hepatocellular carcinoma (liver cancer) exhibits high heterogeneity in its development mechanisms, tissue morphology, tumor microenvironment, and molecular levels. Tumor organoids are miniature 3D tumor cell models derived from the primary tumors of patients and are cultivated in the laboratory. Tumor organoid models can closely mimic the structural and functional characteristics as well as the gene expression profiles of primary tumor tissues, reproducing their heterogeneity. In 2017, Broutier et al. [65] successfully cultivated three subtypes of primary liver cancer organoids that retained the histological structure, gene expression, and genomic profiles of their original tumors. Even after long-term expansion under the same culture conditions, these organoids can differentiate between different tumor tissues and subtypes. Allograft studies demonstrated that derived organoids preserved their tumorigenic potential, histological features, and metastatic properties in vivo. These organoids have been utilized in biomarker identification and drug screening tests, leading to the discovery of the potential therapeutic drug SCH772984, an ERK inhibitor, for primary liver cancer. 2022, Narayan et al. [66] established a series of organoids derived from 21 patient samples, including 12 from metastatic tumors, three from liver tumors, and six from adjacent nontumor liver tissue. These patient-derived primary liver cancer organoids recapitulated the histological morphology, immunohistochemistry, and transcriptome of the patient’s tumor. These tumor-derived organoids hold significant biomedical potential for understanding liver cancer biology and developing personalized treatment approaches.

## Application of liver organoids

**Fig. 3:**
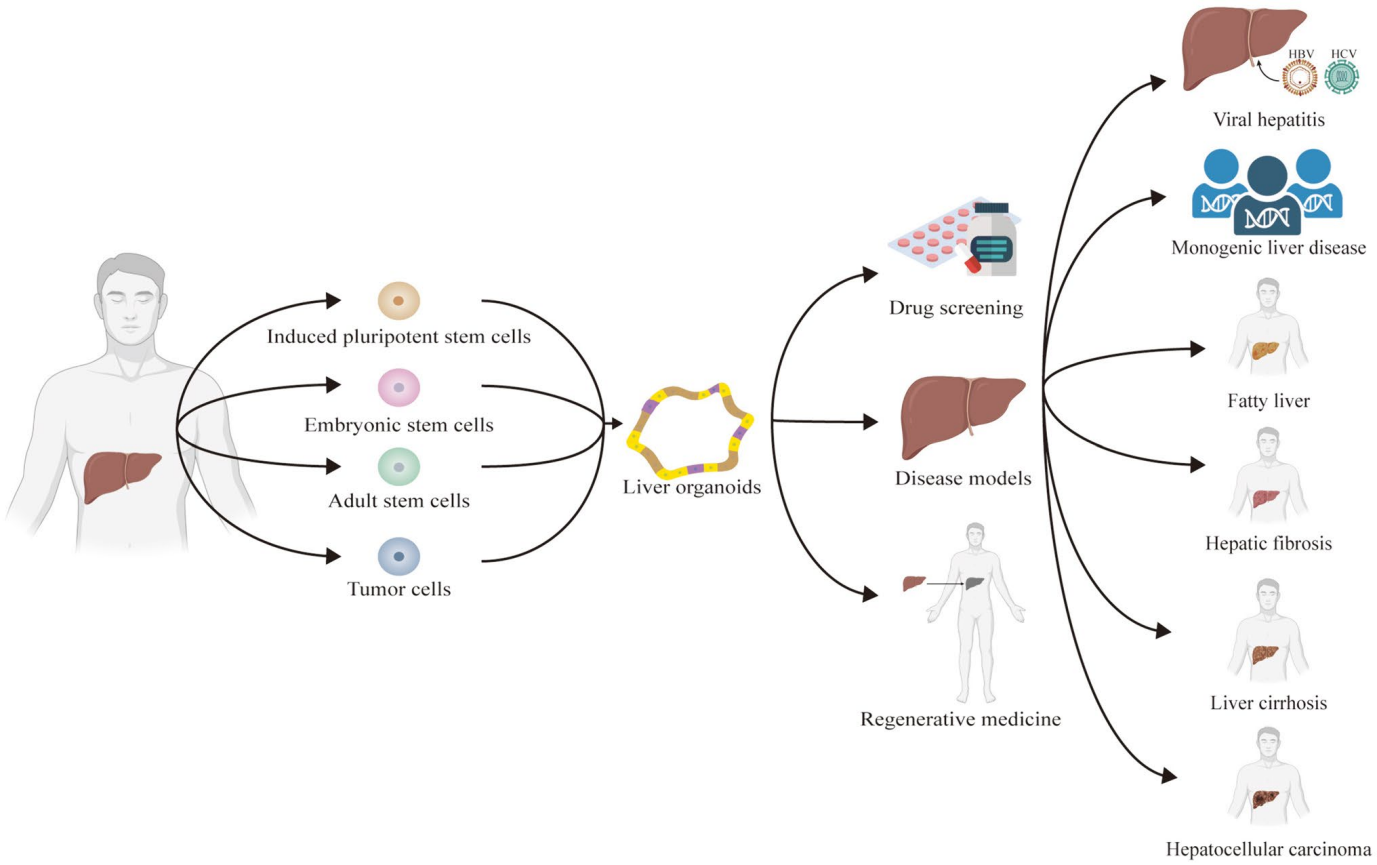
The development and application of liver organoids

Although the application of organoid technology in the research community is still in its early stages, as a novel tool, organoid technology has enormous potential in the fields of disease pathology, cell biology, precision medicine, regenerative medicine, and drug toxicity and efficacy testing (Fig. 3).

### Disease model

Various liver-related diseases can cause liver dysfunction, including viral hepatitis, monogenic liver disease, alcohol-related liver disease (ALD), nonalcoholic fatty liver disease (NAFLD), primary hepatic carcinoma (PHC), hepatic fibrosis (HF), and liver cirrhosis. Multiple liver disease organoid models have been established, providing a broad platform for studying the pathogenesis and treatment of liver-related diseases.

#### Viral hepatitis models

Viral hepatitis is an inflammatory liver disease caused by viral infection, with five main types, among which hepatitis B virus (HBV) and hepatitis C virus (HCV) are the most common, both primarily transmitted through bodily fluids [67]. Due to the narrow species tropism exhibited by hepatitis virus, infecting mainly humans and higher primates, suitable animal models are lacking, hindering the development and clinical testing of antiviral drugs [68], although human hepatocellular carcinoma cell lines (such as Huh7 [69], HepG2 [70], and HepaRG [71]) have been utilized to establish models of hepatitis virus infection, but prolonged extensive culturing of these 2D cancer cell lines harbors significant genetic, epigenetic, and functional alterations, severely compromising the fidelity of recapitulating virus-host interactions and evaluating antiviral drug efficacy. The establishment of human liver organoid infection models provides a unique opportunity to circumvent these contemporary challenges.

In 2018, Baktash et al. [72] used single-particle imaging techniques to reveal the sequential events that occur as HCV enters human liver cancer organoids, where HCV initially binds to early receptors recruited to the basal membrane, then aggregates at junctions in a myosin-dependent manner, and finally internalizes into host cells assisted by the epidermal growth factor receptor (EGFR). In the same year, Nie et al. [73] utilized human induced pluripotent stem cells (hiPSCs) to generate a functional liver organoid that inherited the genetic background of the donor. The functional liver organoids showed increased susceptibility to HBV infection and could sustain HBV transmission and produce infectious virus for a prolonged period of time. Furthermore, the study found that viral infection led to liver dysfunction in the functional liver organoids, manifested by downregulation of liver gene expression, release of early acute liver failure markers, and alterations in liver ultrastructure. This provides a promising individualized infection model for the development of personalized therapies for hepatitis. In 2022, Li et al.[74] successfully generated intrahepatic cholangiocyte organoids (ICOs) and hepatocyte organoids from fetal and adult human liver tissues. It was demonstrated that organoids derived from fetal and adult liver were permissive to hepatitis E virus (HEV) replication and supported the entire lifecycle of infection and found that both undifferentiated cholangiocyte organoids and differentiated hepatocyte organoids were susceptible to HEV, which is consistent with clinical observations. In 2023, Meyers et al. [75] demonstrated that HCV can survive and replicate abundantly within human liver organoids, maintaining a mild infection state for several months.

#### Monogenic liver disease models

Liver dysfunction diseases, apart from those caused in part by chromosomal abnormalities or mutations in multiple gene loci, are largely attributable to mutations in single genes. Examples include Wilson’s disease (WD), alpha-1 antitrypsin deficiency (AATD), Wolman’s disease (lysosomal acid lipase deficiency, LAL-D), citrullinemia type 1 (CTLN1), cystic fibrosis (CF), and others [76].

##### Wilson’s disease models

Wilson’s disease (WD) is a hereditary metabolic disorder caused by mutations in the ATP7B gene [77]. The pathogenic gene ATP7B encodes a copper-transporting p-type ATPase, and the loss of function of the copper transporter ATPase leads to impaired copper excretion into the bile. Accumulation of excess free copper can result in cellular toxicity, sustained liver cell damage, and consequent chronic hepatitis, making patients prone to developing cirrhosis and liver cancer [78]. Currently, liver transplantation is the primary treatment for the clinically diverse phenotypes of WD, but timely diagnosis remains a major challenge. In order to better study WD, in 2015, Nantasanti et al. [79] have established a canine liver-like organoid model with a defect in the COMMD1 gene, mimicking the phenotypes of copper storage diseases in humans. This canine liver-like organoid exhibits a tubular structure similar to bile duct organs, and due to the COMMD1 gene defect, a large accumulation of copper ions within the organ cannot be secreted. Overexpression of the COMMD1 gene can restore its function, providing insights into the treatment of copper storage diseases. In 2020, Schene et al. [80] attempted to model monogenic diseases in 3D cultured adult stem cell organoids using a novel primer editing technique, effectively correcting WD caused by mutations in the ATP7B gene. Following the generation of ATP7B knockout (KO) organoids in vitro, it was observed that ATP7B KO organoids were more susceptible to copper-induced cell death compared to organoids with normal ATP7B genotype. Gene editing to correct ATP7B mutations restored copper excretion, indicating that base editing could functionally restore liver organoid mutations in patients with WD. This study confirms the potential of plasmid editing technology in simulating and safely repairing human monogenic diseases, marking an important step towards future clinical applications.

##### Alpha-1 antitrypsin deficiency models

Alpha-1 antitrypsin (AAT) deficiency is a condition characterized by a lack of AAT due to mutations in the SERPINA1 gene [81, 82]. More than 90% of severe deficiency patients are homozygous for Z (Glu342Lys) mutation. AAT in serum is primarily derived from the liver, and in protein misfolding disorders, deficiency of AAT leads to loss of protease function, resulting in damage to target organs. Research on liver diseases caused by AAT deficiency is limited due to the difficulty in obtaining human liver tissue and maintaining primary cultures of human liver cells. In 2015, Huch et al. [83] obtained liver organoid cultures from needle biopsies of homozygous AAT mutation patients, successfully recapitulating the pathological features of AATD in vitro. Clear observation of AAT protein aggregates, similar to those found in original biopsies, was evident in differentiated hepatocytes within the organoids, indicating reduced AAT protease activity. These studies demonstrate that liver organoids carrying different genetic variants of AAT can mimic the specific features of the disease. In 2019, Gómez-Mariano et al. [84] established liver organoid models using liver biopsy tissues from patients with the homozygous, heterozygous, and normal genotypes of SERPINA1 mutations. In this model, liver organoids exhibited high expression of hepatocyte markers. Liver organoids carrying SERPINA1 mutations closely resembled patient liver tissue in terms of gene expression and protein secretion. In 2023, Pérez-Luz et al. [85] assessed lipid accumulation in HepG2 cells overexpressing Z-AAT as well as in patient-derived liver organoids from heterozygous and pureblooded individuals by oil-red staining and evaluated mass spectrometry-based lipidomic analysis and transcriptomic analysis. Based on transcriptomic analysis, pure heterozygous organoids possess many alterations in genetic and cellular processes of lipid metabolism with specific effects on endoplasmic reticulum, mitochondrial and peroxisomal dysfunction. The relationship between intrahepatic accumulation of Z-AAT and alterations in lipid homeostasis was revealed. These studies suggest that liver organoids provide an excellent model for studying liver diseases associated with mutations in the SERPINA1 gene.

##### Citrullinemia type 1 models

CTLN1 is an autosomal recessive urea cycle disorder caused by mutations in the ASS1 gene, resulting in a deficiency of argininosuccinate synthetase (ASS), characterized by hyperammonemia leading to neurological damage [86]. In the treatment of CTLN1, besides conventional therapeutic approaches, organoid technology has also been employed to simulate liver disease progression, drug screening, and personalized therapy. In 2019, Akbari et al. [87] utilized EpCAM-positive endothelial cells differentiated from iPSCs as intermediates to generate a long-term expandable hepatic organoid culture system that retains the ability to differentiate into mature hepatocytes. They further developed patient-specific iPSCs for CTLN1 and differentiated them into hepatic organoids, finding that CTLN1 organoids exhibited normal morphology, differentiation potential, and metabolic function but showed excessive ammonia accumulation. Overexpression of the ASS1 gene could reverse this phenotype.

##### Wolman’s disease models

Wolman’s disease is a type of lysosomal acid lipase (LAL) deficiency, characterized by a rare disorder of lysosomal lipid storage and complete or partial absence of LAL activity due to mutations in the LIPA gene encoding LAL, ultimately leading to lipid accumulation, hepatomegaly, and hepatic dysfunction [88]. In 2019, Ouchi et al. [89] used iPSCs to establish liver organoid models containing hepatocytes, stellate cells, and Kupffer cells. They discovered that liver organoids derived from iPSCs of patients with LIPA deficiency exhibited a greater propensity for lipid accumulation and higher degrees of fibrosis. However, treatment of these liver organoids with a farnesoid X receptor (FXR) agonist could alleviate the phenotypes of lipid accumulation and fibrosis. This research indicates that liver organoids represent a valuable system for testing drug efficacy and providing personalized drug discovery.

##### Cystic fibrosis models

Cystic fibrosis (CF) is an autosomal recessive genetic disorder caused by mutations in the CFTR gene [90]. CFTR, serving as a transmembrane conductance regulator in CF, primarily functions to regulate cAMP-dependent chloride ion channels, mediating the efflux of chloride ions into the bile duct lumen [91]. Mutations in the CFTR gene affect the ability of bile duct cells to transport ATP and chloride ions, resulting in decreased bile flow, reduced bile volume, and further leading to damage, inflammation, and fibrosis in liver cells [92]. In 2015, Ogawa et al. [93] utilized functionally impaired human pluripotent stem cells (hPSCs) derived from patients with CF to generate cystic and/or ductal structures expressing mature bile markers, including apical sodium-dependent bile acid transporter, secretin receptor, cilia, and CF transmembrane conductance regulator (CFTR). They observed that organoids carrying CFTR mutations exhibited characteristics typical of CF patients, such as impaired chloride ion channel function and disrupted cyst formation. Treatment with CFTR agonist VX-770 and cAMP agonist Forskolin could rescue the phenotype of organoids induced by CFTR mutations, indicating the crucial value of bile duct organoids in assessing drug efficacy. In 2021, Amarachintha et al. [94] generated bile duct-like organoids from liver biopsies of infants with biliary atresia. In the biliary atresia organoids, bile duct-like cells exhibited a fundamental mislocalization of cell nuclei, expressed fewer developmental and functional markers, and displayed misplacement of cilia, resembling the clinically diagnosed delayed development of biliary epithelium. Through detailed molecular and functional phenotyping of the organoids derived from liver biopsies, molecular and functional evidence of delayed epithelial development in biliary atresia was revealed. These studies demonstrate that organoids derived from patients may serve as powerful tools for future studies of the pathogenesis of biliary atresia disease and mechanisms of drug action.

#### Alcohol-related liver disease models

Alcohol-related liver disease (ALD) is a leading cause of chronic liver disease worldwide, progressing from alcoholic hepatitis (AH) and hepatic fibrosis (HF) to cirrhosis and hepatocellular carcinoma. Excessive alcohol consumption is the main cause of ALD, and the rate of alcohol consumption in China is increasing annually [95]. Alcohol has become the second-largest etiological factor of liver injury after viral causes [96, 97]. Although abstinence from alcohol remains the main therapeutic intervention to control ALD, there are currently no approved drugs for its treatment [98, 99]. Despite in-depth research on the pathogenesis of ALD, there is currently no known alcoholic liver model that fully recapitulates the entire progression of human ALD, which severely limits our understanding and research progress on ALD [100]. In 2019, Wang et al. [56] developed a derivative model using ESCs that simulated the pathophysiological changes associated with alcohol-induced liver disease, providing a reliable and practical in vitro system for pathophysiological modeling of ALD. Currently, there is a lack of comprehensive research on organoid models of ALD. Most studies have utilized ethanol treatment to induce the progression of normal differentiated liver organoids into ALD organoids. The advantage of this method is its simplicity and convenience, but it is unknown if the induced process accurately represents in vivo development. ALD progression involves the interaction of multiple cell types within the body and is influenced by genetic, epigenetic, and environmental factors [101]. An effective model is crucial for studying the pathogenic mechanisms at different stages of ALD and for increasing the likelihood of discovering new therapeutic targets. Furthermore, researchers have incorporated human embryonic liver mesenchymal cells into liver organoids, developing a model that mimicked the pathological and physiological changes associated with alcoholic liver disease under ethanol treatment. Their study indicated that ESC-derived liver organoids have significant potential as a novel in vitro pathophysiological model for studying alcoholic liver disease[56].

#### Nonalcoholic fatty liver disease models

Nonalcoholic fatty liver disease (NAFLD) progresses from hepatic steatosis to nonalcoholic steatohepatitis (NASH), hepatic fibrosis, cirrhosis, and hepatocellular carcinoma. Currently, liver biopsy remains the gold standard for assessing histological changes in the liver tissue [102]. NAFLD has become the most common cause of liver disease, correlating with epidemics of obesity, type 2 diabetes, and metabolic syndrome [103]. Although therapies to reduce hepatic steatosis are known, the main challenge lies in reversing the inflammatory components in the liver [104]. Currently, there is no effective treatment available to reduce liver inflammation [105]. Therefore, the development of valid models related to NAFLD is crucial for studying its pathogenesis and for developing effective therapeutic drugs, with a focus on reversing inflammation and fibrosis. In 2017, Bossche et al. [106] incubated liver organoids from diseased and healthy control dogs with palmitic and oleic acids to investigate the mechanisms of fatty liver disease. They compared liver lipid accumulation, gene expression analysis, and HPLC–MS of neutral lipids and phospholipids in Extrahepatic Portosystemic Shunt (EHPSS) and Intrahepatic Portosystemic Shunt (IHPSS) dogs with healthy control dogs, suggesting that fatty liver disease may be a consequence of portosystemic shunting, providing a model to better understand fat accumulation in NAFLD. In 2020, Elbadawy et al. [107] generated liver organoids from mice with mild (NASH A), moderate (NASH B), and severe (NASH C) nonalcoholic steatohepatitis induced by methionine- and choline-deficient diets. This model replicated the characteristics of NASH liver tissue and could be used to study genetic stability and lipid metabolism during NAFLD/NASH transition. In 2023, Hendriks et al. [108] used human fetal liver cell organoids to simulate the stages of hepatic steatosis in NAFLD under three different triggers, and through CRISPR that reducing the expression of the fatty acid desaturase 2 (FADS2) gene, which is associated with the risk of nonalcoholic fatty liver, can decrease liver fat accumulation. These organoid models contribute to the study of the pathogenesis of lipid degeneration and identification of drug targets.

#### Hepatic fibrosis and liver cirrhosis models

Chronic liver inflammation can lead to the development of fibrosis, cirrhosis, and hepatocellular carcinoma. Liver fibrosis is characterized by excessive deposition of extracellular matrix (ECM) components, such as collagen and glycoproteins, replacing damaged normal tissue and forming fibrotic scars [109], whereas advanced cirrhosis represents distortion of the liver structure and blood flow [110]. Liver fibrosis, a common pathological stage in chronic liver disease, is a prerequisite for the development of cirrhosis and hepatocellular carcinoma, making it a major global concern. Despite the high prevalence and destructive nature of fibrosis and cirrhosis, effective treatment methods are currently lacking [111], and current approaches mostly focus on alleviating chronic stress [112]. Prevention, treatment, and reversal of liver fibrosis are key to the successful management of chronic liver injury. Understanding the mechanisms underlying liver fibrosis is clinically significant for the development of therapeutic drugs and overall improvement of treatment methods. Therefore, studying the pathogenesis of liver fibrosis, developing therapeutic drugs, and constructing liver fibrosis models with similar pathogenic mechanisms are of great importance [113].

In 2016, Leite et al. [15] used HepaRG cells and primary human hepatic stellate cells (HSCs) to cultivate 3D liver organoids in which the metabolic capacity of the organoids could be activated in a drug- and hepatocyte-dependent manner. After single or repeated exposure to pro-fibrotic compounds, such as allyl alcohol and methotrexate, for 14 days, the liver organoids exhibited fibrosis features, including HSC activation, collagen secretion, and deposition. This novel liver organoid culture model is the first to detect hepatocyte-dependent and compound-induced HSC activation, representing a significant step forward in in vitro compound testing for drug-induced liver fibrosis. In 2021, Guan et al. [114] used CRISPR technology to edit and design liver organoids derived from iPSCs expressing mutations associated with autosomal recessive polycystic kidney disease (ARPKD). These organoids displayed major histological features of ARPKD liver pathology, such as bile duct abnormalities and fibrosis, within 21 days. The transcriptome of ARPKD organoid-derived stellate cells showed similarities with that of common liver fibrosis. This marked the first liver organoid system that mimicked the key features of human liver fibrosis, particularly the excessive assembly of collagen fibers. In addition to gaining deeper insights into the pathogenesis of congenital (or possibly acquired) liver fibrosis, ARPKD organoid tissue can also be used to test the anti-fibrotic effects of potential therapies.

#### Primary liver cancer models

In 2020, primary liver cancer was ranked as the sixth most common cancer and the third leading cause of cancer-related deaths globally [115]. Primary hepatic carcinoma (PHC) is mainly classified into three subtypes: hepatocellular carcinoma (HCC), intrahepatic cholangiocarcinoma (ICC), and combined hepatocellular cholangiocarcinoma (CHC). There are also some rare types of PHC, including fibrolamellar hepatocellular carcinoma (FL-HCC), hepatic angiosarcoma, and hepatic hemangioendothelioma [116]. The etiology and pathogenesis of PHC are not yet fully understood but are likely to be associated with factors such as liver cirrhosis, viral hepatitis, and carcinogenic substances such as aflatoxin and environmental factors. Three-dimensional culture of tumor cells can provide a more accurate representation of tumor cell interactions with the tumor microenvironment, reflecting the entire process of tumor development, and guiding the identification of therapeutic targets and screening of anticancer drugs [117]. Organoids can be generated from induced pluripotent stem cells, embryos or adult, healthy, or diseased tissues. Specifically, liver organoids have been extensively utilized in mechanistic studies aimed at elucidating the molecular pathways underlying PHC development.

In 2018, Nuciforo et al. [118] generated long-term liver organoid cultures from tumor needle biopsies of patients with HCC with different etiologies and tumor stages. The HCC organoids retained the morphological and expression patterns of HCC tumor markers and preserved the genetic heterogeneity of the primary tumors. This study demonstrated that organoid models can be derived from needle biopsy samples of liver cancer and provide a tool for developing personalized therapies. In 2019, Cao et al. [119] cultured 91 organoid lines from 129 liver tissues and tumors. These organoids can be cultured long term in vitro, and approximately 20% of them form tumors when transplanted into immunodeficient mice, confirming their tumorigenic and self-renewal properties. To some extent, these organoids recapitulated the phenotypic heterogeneity, cancer cell composition, and treatment responses observed in liver cancer patients. These model systems offer significant opportunities for advancing liver cancer biology, drug development, and personalized medical research. In the same year, Artegiani et al. [120] used CRISPR/Cas9 technology to induce the loss of function of the ubiquitin protease BAP1 in normal human cholangiocytes in organoids. They found that BAP1 controls the expression of interconnections and cytoskeletal components by regulating chromatin accessibility. Subsequently, further knockout of tumor suppressor genes TP53, PTEN, SMAD4, and NF1 in cultured organoids induces the formation of cholangiocarcinoma. This study demonstrated that the combination of organoid technology and CRISPR/Cas9 provides an experimental platform for investigating the functional mechanisms of cancer genes in humans. In 2022, Lam et al. [121] generated liver organoids from normal human liver tissue and used CRISPR to knock out TP53 and overexpress the R249S mutant to simulate early carcinoma in the human liver. This study revealed that TP53 loss and L3 mutation had distinct tumorigenic effects, jointly conferring early clonal advantage and pro-survival function to normal liver cells. Unlike traditional two-dimensional culture models, three-dimensional liver cancer organoids cultured in vitro can maintain a high proliferative capacity while preserving the properties of the original tumor, providing a convenient, stable, and reproducible experimental model for tumor research.

### Drug screening

Liver organoids can effectively mimic the physiological functions and disease progression of the liver in vitro, making them highly promising for large-scale drug safety assessments and efficacy screening. Drug-induced liver injury (DILI) refers to liver damage caused by the drugs themselves, their metabolites, or decreased tolerance to drugs owing to specific conditions. It is one of the most common causes of acute liver failure and the withdrawal of marketed drugs. Liver organoids serve as reliable in vitro models for predicting drug-induced liver toxicity [122, 123]. In 2013, Kostadinova et al. [124] established an in vitro 3D liver co-culture system that could maintain long-term liver cell functionality and phenotype. This system comprises primary parenchymal and nonparenchymal liver cells, retains the inductive properties of cytochrome P450, forms bile duct-like structures, and responds to inflammatory stimuli, allowing the detection of adverse drug reactions in a physiological context. Studies have shown that liver organoid systems containing multiple cell types and more complex structures can simulate toxic drug responses more accurately. In 2023, Zhang et al. [125] developed a human liver organoid (HLO) chip system using three independent iPSC lines to simulate in vitro liver function and high-throughput identification of hepatotoxic compounds. The study demonstrated that dispersed HLOs from the three iPSC lines had similar DILI prediction capabilities to intact HLOs in high-throughput screening, and observed hepatotoxicity related to telaprevir, similar to the clinical presentation of DILI in patients.

In drug screening and development, traditional preclinical trials primarily rely on cell line cultures and patient-derived xenograft (PDX) models. However, owing to species differences in PDX models and the inability of 2D cell line models to accurately reflect in vivo processes, the obtained experimental results may not be accurate. Liver organoids can be derived from primary cells isolated from patient surgery or biopsy tissues and cultured in a 3D format. They not only exhibit similar structures and functions to primary cell tissues but also maintain the genetic stability of the original tissue during long-term in vitro culture, making them more suitable for high-throughput screening and genetic manipulation. In 2017, Broutier et al. [65] reported for the first time that different types of primary liver cancer organoids exhibit varying sensitivities to different therapeutic drugs and confirmed the potential of the ERK inhibitor SCH772984 as a treatment for PLC. This study demonstrated that liver tumor organoid models can provide personalized treatment options for patients and serve as effective preclinical models for drug screening. In 2019, Li et al. [126] established 27 liver cancer organoid lines from multiple regions of surgical specimens and screened 129 anticancer drugs. The results showed that most of the 129 drugs were either ineffective or active only in a subset of liver cancer organoid lines, with only a few drugs showing at least moderate activity in the majority of liver cancer organoid lines. This study highlights the effectiveness of liver cancer organoid models for preclinical drug screening. Liver cancer organoid models hold significant potential as a novel approach for drug discovery and have significant implications for predicting patient responses to specific drugs. In 2023, Hendriks et al. [108] conducted a screening of 17 candidate drugs for nonalcoholic fatty liver disease and identified compounds that effectively alleviate lipid degeneration.

### Regenerative medicine

Liver transplantation remains the only effective treatment for end-stage liver diseases. However, the shortage of donors and the challenges associated with lifelong immunosuppression greatly limit the application of liver transplantation. In 2013, Takebe et al. [47] reported the successful generation of vascularized and functional human liver organoids from human induced pluripotent stem cells (iPSCs). When these organoids were transplanted into a mouse model of liver injury, they were rapidly engrafted within the mouse liver, leading to improved survival time and rate in the transplanted mice. The successful engraftment of these organoid buds demonstrated the ability of liver organoids to exert certain physiological functions in mice, offering a promising new approach for studying liver transplantation and regenerative medicine. In 2018, Peng et al. [127] discovered that the inflammatory cytokine TNF-α, induced by injury, promoted the expansion of liver cells in 3D culture, allowing for continuous passaging and long-term culture for over 6 months. Furthermore, when these expanded liver cells were transplanted into immunosuppressed mice with injured livers, they proliferated rapidly and exhibited significant engraftment. In 2021, Yang et al. [128]utilized a specific 3D bioprinting process to construct a 3D bioprinted liver using HepaRG cells and Bioink. After seven days of in vitro differentiation, the bioprinted liver displayed widespread liver functionality. When transplanted into a mouse model of immunodeficiency and tyrosinemia type I, liver organoids further matured, showed increased synthesis of liver-specific proteins, significantly improved mouse survival rates, and acquired human-specific drug metabolism activities. These studies demonstrate that 3D bioprinting can be utilized to generate human liver tissues and serve as an alternative transplant source for the treatment of liver diseases.

## Prospect

Liver organoids can be effectively derived from induced pluripotent stem cells, embryonic stem cells, adult stem cells, and tumor cells and exhibit some characteristics of in vivo liver tissue. They closely mimic tissue morphology, gene expression, and other features of the liver, making them highly promising for applications in disease modeling, high-throughput drug screening, safety and efficacy evaluation, and regenerative medicine. Compared to traditional animal models and 2D cell culture systems, organoids offer advantages such as self-organizing structures, stable gene profiles, and ease of manipulation, providing a novel in vitro model for biomedical research and overcoming the limitations of animal models and 2D cell cultures.

**Fig. 4:**
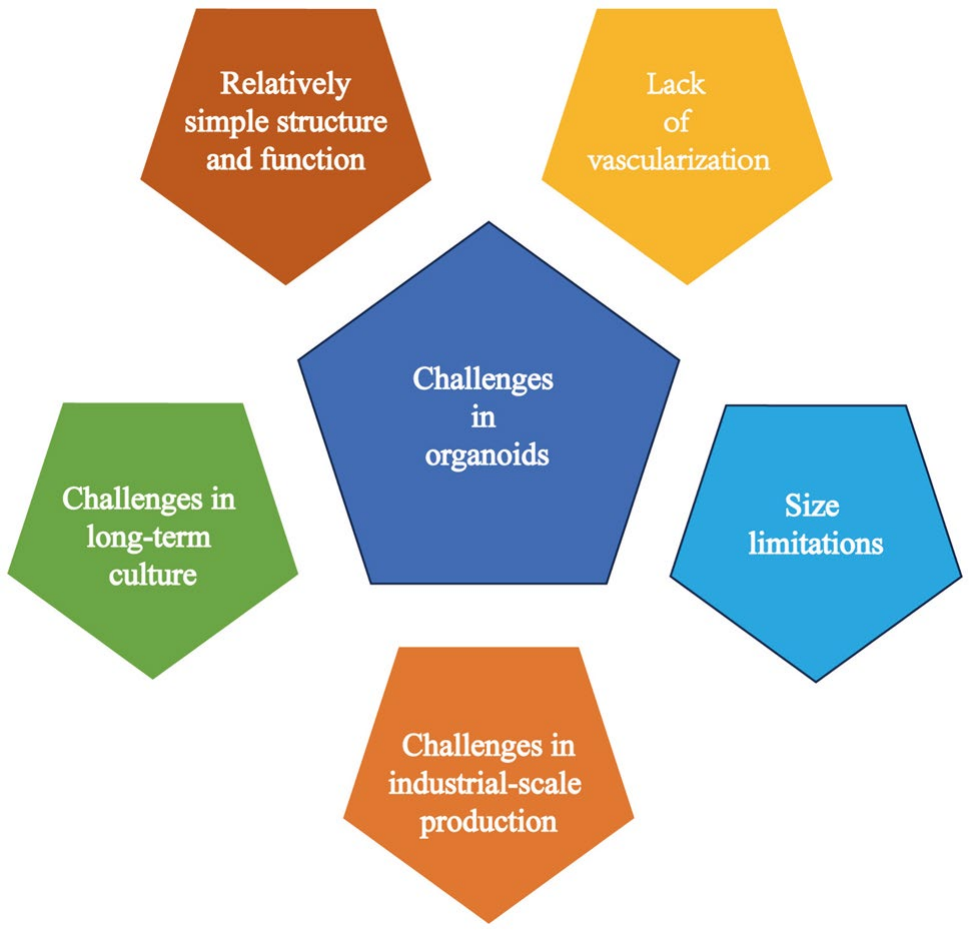
Challenges for organoids

However, this technology is still in its early stages and faces several technical challenges that must be overcome. (1) Integrity of structure and function: Current organoid models often simplify the complexity of real organs, resulting in a relatively simple cellular composition that lacks interactions with other cells in the body microenvironment (such as immune cells and nerve cells). Compared to mature liver tissue, liver organoids cannot fully replicate all cell types, cell–cell interactions, and comprehensive organ functions. Breaking through this bottleneck may perhaps be achieved by developing 3D bioprinting technology to print biocompatible materials and cells with complex structures, mimicking the microenvironment of natural liver tissue [129]. Alternatively, exploring various co-culture systems of cells could better replicate the cellular heterogeneity of the liver. (2) Lack of vascularization: To support the growth and functionality of organoids, they require an effective vascular system. Current techniques still struggle to establish a fully formed and functional vascular network within organoids. However, Skylar-Scott et al. [130] assembled patient-specific organ building blocks (OBBs) derived from induced pluripotent stem cells into living matrices with high cell density. By embedding vascular channels using embedded 3D bioprinting, they successfully constructed perfusable heart organoid. This provides us with a direction. (3) Size limitations: Currently, liver organoids have limited bile duct formation and vascularization. As the size of liver organoids increases, they face challenges in acquiring sufficient nutrients and oxygen and in the efficient removal of metabolic waste. Hypoxia and the accumulation of metabolic waste can lead to cell death and tissue necrosis, thus restricting the growth of liver organoids. (4) Challenges in industrial-scale production: During the cultivation of liver organoids, their self-organizing formation and the achievement of functional and structural maturity involve significant uncertainties, preventing the realization of industrial-scale production. Researching and developing automated and scalable production processes to reduce costs and increase efficiency are important for organoid development. (5) Challenges in long-term culture: With the passage of time, organoids may lose stability or undergo degenerative changes, limiting their application in long-term experiments (Fig. 4).

In the future, with continuous technological advancements and resolution of these challenges, it may be possible to achieve in vitro cultivation of liver organoids incorporating multiple cell types, including vasculature, neurons, and immune cells. This would enable the exploration of interactions between liver organoids and various tissues, leading to broader application and clinical translation of liver organoid technology in drug development, disease modeling, and personalized therapies.

## Data Availability

Not applicable.
